# Why Is Chloroform so Well Borne in Midwifery?

**Published:** 1883-04

**Authors:** Fancourt Barnes


### WHY IS CHLOROFORM SO WELL BORNE IN MIDWIFERY?

It has long been a recognized fact that the administration of chloroform in midwifery is not followed by the deaths which so frequently happen when it is given during surgical operations. No explanation has, so far as I know, yet been given. The explanation, I believe, lies in the condition of the heart and vascular system during pregnancy. The changes undergone by the heart and vascular system during gestation are well known. The heart becomes hypertrophied, the system becomes enlarged by the distention of the existing veins and the development of fresh venules. The quantity of blood is increased. When chloroform produces fatal syncope, it does so by its depressing action of the heart. This is well known. When, however, the heart is sirong, stronger than usual, as in the hyputropical heart of pregnancy, it can more easily withstand the action of chloroform. Is not this the reason that the hypertrophied heart of pregnancy is unaffected by chloroform? I think it is clearly so.—*Fancourt Barnes, M. D., in British Medical Journal.*

